# An Argument for Expanding the Role of Pediatric Decision-Making and Preference in Child Abuse/Neglect Assessments and Plan

**DOI:** 10.1155/2021/9910304

**Published:** 2021-06-18

**Authors:** Rissa Fedora, Becky Li

**Affiliations:** ^1^University Hospital and Medical Center, Tamarac, FL 33321, USA; ^2^Dr. Kiran C. Patel College of Allopathic Medicine, Fort Lauderdale, FL 33328, USA

## Abstract

Child maltreatment can have long-term sequelae and thus requires appropriate interventions. In the United States, reports of suspected child maltreatment are primarily handled by the Child Protective Services (CPS). We present a case of a 12-year-old female who was involuntarily hospitalized for suicidal ideation after CPS responded to a report of her abuse by her mother. Despite continuously expressing fear of her mother and pleading to not be discharged home, CPS ultimately determined that the child was safe to return home to her abuser. The child's subsequent loss to follow-up puts the child's safety and long-term well-being into question. In this article, we discuss the current protocol of CPS reporting, investigation, and outcome. We also explore the roles of pediatric decision-making and forensic or custody evaluation when maltreatment is apparent.

## 1. Introduction

Child maltreatment, consisting of abuse or neglect by a caretaker, often results in increased morbidity and mortality with long-term sequelae. In the United States, Child Protective Services (CPS) is the main organization which responds to reports of suspected child maltreatment. In 2019, CPS identified about 656,000 victims (or approximately 22%) out of 3 million reports. Of the identified victims, 60.8% received CPS postresponse and only 22.9% were removed from their homes [1].

A 2020 Netflix documentary titled *The Trials of Gabriel Fernandez* provides a paradigm of the current process of responding to child maltreatment. In the documentary, multiple reports of suspected physical abuse of an 8-year-old Gabriel were made to CPS. At the end of each investigation, Gabriel's living condition remained unchanged. Gabriel even asked to live with his grandparents, whom he previously resided with for three years. Despite multiple reports and his request to live at his former residence, he was deemed to be safe to return home to his abusers, which eventually resulted in his death.

We present a case of child abuse with similar investigation outcome, where a victim was deemed safe by CPS to return home to her abuser. The objectives of this paper are to discuss the current CPS protocol in response to maltreatment reports and to explore the child's autonomy and role in decision-making regarding placement outcome.

## 2. Case Presentation

A 12-year-old female with no prior psychiatric history was brought involuntarily by police for suicidal ideation with a plan after CPS was notified of suspected emotional and physical abuse by her mother. The child reported that her mother found out she hid food under her bed, which resulted in her mother causing physical harm with an electrical cord and a belt to the child's bilateral upper extremities. After this incident, the child took a knife from the kitchen and hid it in her room, with the intent of harming herself and ending her life. CPS found the knife, resulting in her involuntary psychiatric hospitalization. On evaluation, the child also reported that this was not the first time her mother physically abused her. She also stated her mother often called her derogatory names.

Upon obtaining collateral information from the mother, she admitted to disciplining her daughter. She said she hit her with her bare hand first, but her daughter continued to talk back to her. She then hit her with a belt out of anger. Contrary to the child's statement, the mother claimed it was the first time she hit her daughter with a belt. She also stated that her daughter often verbalized suicidal statements when she did not get her way.

On physical examination, the patient appeared as stated age with fair grooming. Multiple cord-shaped and belt-buckle-shaped bruises were found on the child's arms and legs. She was calm and cooperative although mildly guarded. She presented with poor eye contact and some psychomotor retardation. Her speech was slowed and low in volume. She reported feeling sad and fearful with congruent affect. Her thought process was logical and linear. Thought content included fear of her mother and suicidal intent and plan as above. Insight and judgement were fair and congruent with her developmental age.

The patient endorsed symptoms consistent with major depressive disorder, including depressed mood for about a month, poor appetite, feelings of guilt regarding the abuse, poor concentration reflected in her poor school grades, insomnia, and suicidal ideation with intent and plan. Differential diagnoses included child physical abuse, child psychological abuse, and other unspecified trauma- and stressor-related disorder. The patient was admitted to the inpatient youth psychiatric unit with a plan to improve the patient's mood and suicidality through both pharmacological and nonpharmacological therapy.

The patient was provided with group and individual psychotherapy. Since her mother did not consent to psychopharmacological treatment, no medications were given. Throughout her hospital stay, the child often expressed fear of her mother and pleaded with the hospital staff to not discharge her home. At the end of the investigation, CPS determined that the child was safe to return home. After her mood had improved and she no longer endorsed suicidal ideation, she was discharged home in the custody of her mother. A follow-up appointment with her outpatient psychiatrist was set up upon discharge. The appointment was later rescheduled and resulted in a no-show.

## 3. Discussion

When a suspected maltreatment is reported, CPS screens if there is sufficient evidence to warrant an investigation. Provided that the screening criteria is met, CPS will respond and launch an investigation, potentially interviewing the involved parties. If there is immediate danger to the child, the child may be moved temporarily to another location (i.e., foster home, relative's home) during the ongoing investigation and impending court proceedings. The investigation yields findings on whether maltreatment is substantiated—the definition of which can vary by state [2]. Investigation details and deciding factors are kept confidential within the child welfare system. Despite validation of maltreatment, removing a child from the main caretaker is considered to be the last resort. As mentioned previously, only 22.9% of victims are removed from home as child welfare is aimed at promoting “permanency” and helping families successfully care for their children whenever possible [2]. This favored decision is based on studies suggesting that removal from home can add additional stressors and exacerbate trauma [3]. Placing a child in foster care may result in elimination of secondary support systems and higher rates of delinquency and teen pregnancy [4].

When a child is deemed safe to return home, the family may be provided with in-home services to improve their home situation [4]. Nevertheless, challenges arise when victims express fear and unwillingness to return to their abusers. The loss to follow-up in our case further probes questions regarding the child's safety and long-term well-being. This argues for a child's role in influencing discharge placement. We believe this should be evaluated on a case-by-case basis.

Autonomy is a critical ethical principle that remains controversial in pediatric patients. In the United States, competency to make medical decisions is currently determined by age with the exception of emancipation. However, there are multiple studies indicating that preadolescent children have the capacity to make informed decisions, which involve understanding, appreciating, reasoning, and expressing their choice [5, 6]. In the case of our 12-year-old patient, she was able to communicate her comprehension of placement, perceive outcomes of placement options, weigh the risks and benefits of living in different settings, and express her choice. Both the American Academy of Child and Adolescent Psychiatry and American Medical Association recommend involving youths and obtaining their assent in their decision-making process. If a child refuses to assent, it is recommended to respect their refusal and explore their reasoning.

The complexity of this case lies in the fact that the law currently does not provide children with autonomy to make their own decisions when it comes to placement preferences following validation of maltreatment. Nevertheless, child forensic psychiatrists can serve as advocates and provide supporting evidence that may influence this ultimate decision. A forensic evaluation may be incorporated in child maltreatment cases to assess the child's decision-making capacity and provide directions and guidance of placement decisions. In the event that CPS substantiates a maltreatment claim, a forensic psychiatrist shall conduct evaluation as well as treatment of the child.

Child custody and maltreatment evaluations may be incorporated into cases involving child abuse or neglect, substance abuse, and disagreement over parental custody [7]. Forensic evaluators meet with each parent or caregiver separately, together with the child, and the child alone [7]. Collateral information may also be obtained from extended family and other sources [8]. A series of psychological tests are performed to assess parental emotional well-being, personality, and attitude [7]. Regarding child maltreatment, forensic psychiatrists can provide guidance concerning placement, present their recommendation on terminating parental rights, and testify in court or at depositions as necessary [7].

Furthermore, psychiatric treatment of the abused or neglected children should be mandated as in order to prevent long-term negative impact. Current examples of successful prevention and treatment programs only address parents and their issues that interfere with successful parenting [9]. Although such programs are necessary, it is also essential to ensure the children's well-being through ongoing psychiatric care. Forensic child psychiatrists shall provide continuous assessments of mood and suicide risk while home-based evaluation programs assure compliance of outpatient psychiatric follow-ups [10].

## Data Availability

Data is not applicable. No datasets were generated or analyzed.
